# UK Medical Cannabis Registry: An Analysis of Outcomes of Medical Cannabis Therapy for Hypermobility‐Associated Chronic Pain

**DOI:** 10.1002/acr2.70024

**Published:** 2025-03-13

**Authors:** Mary Dickinson, Simon Erridge, John Warner‐Levy, Evonne Clarke, Katy McLachlan, Ross Coomber, Wendy Holden, James J. Rucker, Michael W. Platt, Mikael H. Sodergren

**Affiliations:** ^1^ Medical Cannabis Research Group, Imperial College London London United Kingdom; ^2^ Medical Cannabis Research Group, Imperial College London, and Curaleaf Clinic London United Kingdom; ^3^ Curaleaf Clinic London United Kingdom; ^4^ Curaleaf Clinic and St. George's Hospital NHS Trust London United Kingdom; ^5^ Curaleaf Clinic, Kings College London, and South London & Maudsley NHS Foundation Trust London United Kingdom

## Abstract

**Objective:**

The study aims to evaluate the clinical outcomes in patients with hypermobility spectrum disorder (HSD) and hypermobile Ehlers–Danlos syndrome (hEDS) with chronic pain following treatment with cannabis‐based medicinal products (CBMPs).

**Methods:**

This was a case series conducted with the UK Medical Cannabis Registry. The primary outcomes were changes in the following validated patient‐reported outcome measures at 1, 3, 6, 12, and 18 months compared with baseline: Short‐Form McGill Pain Questionnaire 2 (SF‐MPQ‐2), pain visual analog scale score (Pain‐VAS), Brief Pain Inventory (BPI), five‐level EQ‐5D (EQ‐5D‐5L), Single‐Item Sleep Quality Scale (SQS), General Anxiety Disorder Seven‐Item Scale (GAD‐7), and Patient Global Impression of Change. The incidence of adverse events was analyzed as secondary outcomes. Statistical significance was defined as *P* <0.050.

**Results:**

A total of 161 patients met inclusion criteria. Improvements were observed in BPI severity and interference subscales, SF‐MPQ‐2, and Pain‐VAS (*P* < 0.001). Changes were also seen in the EQ‐5D‐5L index value, SQS, and GAD‐7 (*P* < 0.001). A total of 50 patients (31.06%) reported one or more adverse event with a total incidence of 601 (373.29%). The most frequent rating for adverse events was moderate (n = 258; 160.25%), with headache being the most common (n = 44; 27.33%).

**Conclusion:**

An association was identified between patients with HSD/hEDS with chronic pain and improvements in pain‐specific and general health‐related quality of life following the commencement of CBMPs. CBMPs were also well tolerated at 18 months. These findings must be interpreted within the context of the limitations of study design but add further weight to calls for randomized controlled trials.

## INTRODUCTION

Hypermobility spectrum disorders (HSDs) describe a group of poorly recognized connective tissue conditions, characterized by joint instability and subsequent chronic pain.^1^ Manifestations of HSD range from asymptomatic hypermobility to generalized hypermobility affecting multiple joints with subluxations and dislocations.^2^ Similarly, hypermobile Ehlers–Danlos syndrome (hEDS) is characterized by generalized joint hypermobility, musculoskeletal manifestations, and mild skin hyperextensibility.^3^ Due to the overlap in clinical and molecular characteristics for HSD and hEDS, they are often considered together.^4^

Determining the precise prevalence of HSD/hEDS has proved difficult with changes in categorization and the absence of definitive diagnostic tests. A recent UK symptom‐based survey estimated HSDs were present in 3% of the general population.^2^ Furthermore, hypermobility is more common and severe in women and children, suggesting both age and gender specificity.^5^, ^6^

Chronic pain is a major problem faced by individuals with HSD/hEDS and, for many patients, acts as the primary incentive for seeking care.^7^, ^8^ Individuals affected by acute joint pain following initial subluxation or dislocation may develop a combination of nociceptive, neuropathic, and nociplastic chronic pain.^7^, ^9^ Current management of chronic pain requires a multidisciplinary approach, incorporating physical and talking therapies, alongside medication.^10^ Yet, a paucity of research on the underlying mechanisms and treatment of pain in both HSD and hEDS means current pain management often proves inadequate, with limited research on effective treatment options.^8^, ^9^ If left unattended or insufficiently managed, pain limits functionality and all aspects of mental, physical, social, and economic well‐being.^10^

Cannabis‐based medicinal products (CBMPs) have emerged as a potential alternative for chronic pain management, acting on the endocannabinoid system (ECS), which plays a pivotal role in pain regulation.^11^ The ECS is composed of cannabinoid receptors and their endogenous ligands, which stretch throughout the peripheral and central nervous systems.^12^ Within cannabis there are numerous cannabinoids, among which cannabidiol (CBD) and (−)‐trans‐Δ^9^‐tetrahydrocannabinol (THC) are the most extensively researched.^13^ Although cannabis is classified as a class B controlled drug in the United Kingdom, CBMPs are legal if prescribed by specialist practitioners.^14^, ^15^ CBMPs are regulated under strict legislation, requiring UK Home Office licensing for production, import, or supply in most cases.^14^, ^15^ Although pure CBD is not controlled, most products are subject to the same regulations due to trace amounts of controlled THC.^14^, ^15^ Several licensed suppliers provide CBMPs across the United Kingdom in compliance with the regulatory standards.

Although some meta‐analyses suggest that CBMPs are associated with improvements in pain severity, physical functioning, and sleep quality in individuals with chronic pain, there is a lack of consensus on their efficacy.^11^, ^12^, ^15^, ^16^ The International Association for the Study of Pain (IASP) conducted an overview of systematic reviews, concluding that most are of poor quality.^17^ The IASP subsequently conducted their own systematic review, finding that the evidence on CBMPs’ efficacy was equivocal.^18^ However, this systematic review relied upon a mixed collection of acute and chronic pain, including 10 studies with follow‐ups at earlier than one week across 12 different pain conditions.^18^ A subsequent meta‐analysis conducted by Wang et al^16^ modeled a 10% risk difference in reporting a clinically significant improvement in chronic pain. To conduct this analysis, however, the study authors had to combine the reported effects of different CBMPs, which were limited to noninhaled formulations.^16^ The reason for this lack of consensus is the underlying heterogeneity and low quality of studies that have sought to evaluate CBMPs for chronic pain.

In HSD‐/hEDS‐associated chronic pain, there have been no randomized controlled trials conducted to date. Yet, existing evidence suggests it may have therapeutic potential. A qualitative investigation into complementary medicines reported by patients with hEDS in the United States indicated 48% of the participants had already trialed one or more formulations of cannabis and reported pain‐relieving effects.^19^ Moreover, a study from a French hospital found over 10% of studied patients with hEDS reported regular cannabis consumption for pain relief.^20^ A case study from the United Kingdom of a patient with hEDS who was prescribed a combination of oil and dried flower CBMPs had similarly highlighted a reduction in pain after starting the medication.^21^ Within three months, this was accompanied by elimination of prescribed opioids from 220 mg oral morphine equivalents per day at baseline, increased mobility, and reduction in care needs.^21^

Although emerging preclinical and clinical evidence supports the potential of CBMPs for chronic pain management, most investigations specifically for the treatment of HSD/hEDS rely on qualitative and anecdotal evidence with modest sample sizes. The limited research addressing chronic pain linked with hypermobility reflects a largely neglected area within the field of medicinal cannabis therapy. The UK Medical Cannabis Registry (UKMCR) was developed to help bridge the evidence gap between current clinical practice and the paucity of high‐quality randomized controlled trials on CBMPs by providing the largest real‐world data repository on patient outcomes in the United Kingdom.^22^, ^23^, ^24^ Studies evaluating the outcomes of patients with chronic pain from the UKMCR have found improvements in pain severity and interference alongside symptoms of generalized anxiety and poor sleep.^25^, ^26^ However, these have assessed outcomes across a heterogenous selection of chronic pain etiologies. The primary aim of the present study was to assess changes in pain severity specifically in individuals with HSD‐/hEDS‐associated chronic pain. Secondary outcomes included changes in health‐related quality of life (HRQoL) and the incidence of adverse events (AEs) in this population.

## PATIENTS AND METHODS

### Study design

This study is a case series evaluating the outcomes of individuals with HSD‐/hEDS‐associated chronic pain who were prescribed CBMPs. The primary objective was to assess changes in pain‐specific patient‐reported outcome measures (PROMs) from baseline to follow‐up. Secondary outcomes included changes in HRQoL and the incidence of AEs.

### Setting and participants

Data were extracted from the UKMCR, established by the Curaleaf Clinic in 2019. The registry, the largest pseudonymized database of patients with CBMP use in the United Kingdom and Channel Islands, has previously been used to analyze outcomes in patients with various chronic pain conditions.^25^, ^26^ Patients enrolled in the registry complete baseline and follow‐up questionnaires, allowing for longitudinal assessment of treatment outcomes.^22^

All medications were prescribed in accordance with UK laws and regulations. CBMPs have been legally available on prescription since November 2018.^27^ They are only permitted to be prescribed to individuals who have failed to respond to licensed treatments for their condition. The decision to initiate a patient on CBMP treatment can only be made by a specialist in the treatment that is being sought and must be supported by a multidisciplinary team of peers from other specialities.^27^ Physicians are free to prescribe a range of CBMPs from licensed producers who import CBMPs into the United Kingdom that meet the standards set by the Medicines and Healthcare Regulation Agency.^28^ At the time of the present analysis, the product formats included medium‐chain triglyceride oils (isolated phytocannabinoids or full‐spectrum products) and dried flower. Oils are administered sublingually or orally, whereas dried flower is vaporized and inhaled.

Baseline demographic and clinical data were recorded at the initial consultation. Patients were allocated a primary indication for treatment with CBMPs and additional secondary or tertiary diagnoses if present. Reporting of AEs is completed at the time of incidence or concurrently with PROMs, using a bespoke online platform, or at follow‐up appointments if left unreported. Inclusion criteria extended to individuals with a primary diagnosis of HSD‐/hEDS‐related chronic pain. Patients were required to have completed at least one baseline PROM assessment and enrolled in the UKMCR at least 18 months before the date of data extraction (December 13, 2023).

All participants provided informed written consent before consecutive enrollment in the study. Ethical approval for the UKMCR has been provided by the Central Bristol Research Ethics Committee (22/SW/0145). Reporting for the present study has followed Strengthening the Reporting of Observational Studies in Epidemiology (STROBE) guidelines.^29^

### Data collection

Initial baseline demographic data were gathered during the first consultation, including age, gender, medical history, height, and weight. Occupation was recorded using the International Standard Classification of Occupations.^30^ The primary reason for CBMP treatment was documented alongside other relevant diagnoses and comorbidities. The Charlson Comorbidity Index was subsequently calculated to determine the relative health status of this population.^31^

Patient tobacco, alcohol, and cannabis consumption were recorded at baseline through smoking status, pack‐year history, weekly alcohol consumption (units), cannabis use status, frequency of cannabis use, route of cannabis consumption, and prior cannabis use in gram‐years. At the initial assessment and subsequent follow‐ups, CBMP prescription administration route, cannabinoid contents, and dose were reported. All prescriptions met the standards of good manufacturing practice (GMP).^32^ The concentration of active pharmaceutical ingredients included in this analysis is that provided on the prescription and by the manufacturer. However, in accordance with GMP, there is up to 10% permitted variance in active ingredients, including CBD and THC. For dried flower, the dose was calculated by multiplying the prescribed daily quantity (in grams) by the concentration of each major cannabinoid in each product (in milligrams per gram).

Data collection was completed remotely through online PROMs and AE questionnaires at 1, 3, 6, 12, and 18 months after treatment commencement. All participants were asked to complete validated pain‐specific and general HRQoL PROMs.^28^, ^29^ PROMs were collected via a bespoke data collection platform designed to help optimize data collection. In an evaluation of this data collection method, 91.6% and 73.1% of participants found completing PROMs and AEs easy through this platform.^33^

### Outcome measures

The subsequent PROMs were documented at the initial assessment and at each follow‐up interval for all patients: the Short‐Form McGill Pain Questionnaire 2 (SF‐MPQ‐2), a pain visual analog scale score (Pain‐VAS), Brief Pain Inventory (BPI), five‐level EQ‐5D (EQ‐5D‐5L), Single‐Item Sleep Quality Scale (SQS), and General Anxiety Disorder Seven‐Item Scale (GAD‐7). The Patient Global Impression of Change (PGIC) was additionally recorded at each follow‐up interval but not at the baseline.

### Pain‐specific PROMs

The SF‐MPQ‐2 evaluates the severity and characteristics of chronic pain.^28^ It uses 22 descriptors to assess pain across four major domains: continuous, intermittent, neuropathic, and affective. Each descriptor is rated on a scale of 0 (no pain) to 10 (worst pain). The overall pain score is calculated as the mean of all 22 categories.^34^, ^35^ The minimal clinically important difference (MCID) of the overall score is defined as a one‐point improvement.^36^ The Pain‐VAS allows the assessment of perceived pain intensity in which individuals indicate a point on a 10‐cm line corresponding to their current pain intensity, from 0 (no pain) to 10 (worst pain).^37^, ^38^ The MCID of the Pain‐VAS is 1 cm.^39^, ^40^ The BPI is a self‐reported two‐part scale that measures pain severity and interference. It includes 11 categories for scoring both pain severity and interference on a scale of 0 (no pain/no interference) to 10 (worst pain/complete interference). The MCID of each subscale is defined as a one‐point improvement.^40^

### HRQoL‐related PROMs

The EQ‐5D‐5L is a self‐reported scale evaluating general HRQoL across five domains (mobility, self‐care, usual activities, pain/discomfort, and anxiety/depression). Individuals rate their quality of life from 1 (no problems) to 5 (extreme problems) for each of the domains.^41^ The severity ratings and domains are combined to generate a five‐digit code corresponding to the UK EQ‐5D‐5L index values, ranging from <0 (health status worse than death) to 1 (optimum health).^42^ The SQS questionnaire measures sleep quality over the previous week from 0 (terrible) to 10 (excellent).^43^ The MCID is defined by an improvement of 2.6 or greater. The GAD‐7 screens for and assesses the severity generalized anxiety disorder (GAD) symptoms. Individuals report how frequently they have experienced the seven core symptoms of GAD in the last two weeks from 0 (not at all) to 3 (nearly every day).^44^ Each answer receives a score corresponding to the frequency rating (0–3). The scores are summed to produce an overall score from 0 to 21, with ≥5, ≥10, and ≥15 signifying mild, moderate, and severe anxiety symptoms, respectively.^44^, ^45^ The MCID is a reduction of four points or more.^46^ The PGIC reflects the perception of each individual for their overall improvement following the commencement of treatment. At each follow‐up, individuals rate their change across seven points from no change to a considerable improvement.^47^

### AEs

AEs were collected through self‐reporting, direct questioning by researchers, or documentation by clinicians. AEs were classified according to the Common Terminology Criteria for Adverse Events version 4.0 and categorized by severity (mild, moderate, severe, and life‐threatening/disabling).^48^

### Missing data

The baseline‐observation‐carried‐forward (BOCF) approach was implemented to accommodate for missing PROM data from follow‐ups. This measure biases results to have no positive benefit and no significant assumptions on the data following treatment.^49^, ^50^

### Statistical methods

Descriptive statistics were calculated to evaluate patient demographics, medical history, drug and alcohol data, CBMP prescriptions, and AEs. Reporting was presented as the mean ± SD, median, and interquartile range or frequency (in percentage) when suitable. A repeated‐measures one‐way analysis of variance (ANOVA) was performed to analyze the longitudinal changes in PROMs. Pairwise *t*‐tests with Bonferroni correction were performed on variables that were statistically significant on repeated measures (ANOVA).

A univariable logistic regression was conducted to analyze variables associated with a change corresponding with the MCID in the severity subscale at 18 months. These variables were subsequently taken forward into a multivariable logistic regression model. The results of these are presented as odds ratios (ORs) and 95% confidence intervals (CIs). *P* <0.05 was considered statistically significant. All analyses were conducted using RStudio.

## RESULTS

### Patient data

On December 13, 2023, 19,763 patients had enrolled in the UKMCR. After excluding those who had not completed any baseline PROMs, 18,658 participants remained. A total of 4,974 participants received their first prescription on June 13, 2022, or earlier. A total of 161 patients with HSD‐/hEDS‐associated chronic pain were included in the analysis. The BOCF method was used to impute missing data from incomplete PROMs at 1 month (n = 24; 14.91%), 3 months (n = 38; 23.60%), 6 months (n = 59; 36.65%), 12 months (n = 75; 46.58%), and 18 months (n = 99; 61.49%).

### Baseline demographics

The mean age of the cohort was 37.42 ± 10.53 years, and 80.75% (n = 130) identified as women (Table 1). The mean body mass index was 27.19 ± 7.10 kg/m^2^. Half of the participants (n = 82; 50.93%) were unemployed at baseline. Most participants reported prior (n = 20; 12.42%) or current (n = 80; 49.69%) cannabis use, whereas 51.55% (n = 83) had never smoked tobacco (Table 2). Median lifetime cannabis use in current and previous consumers was 4.00 (1.00–15.00) gram‐years. The number of current tobacco smokers was 25 (15.53%). Median weekly alcohol consumption across the patient cohort was 0.00 (0.00–2.00) units.

**Table 1:** Demographic characteristics of participants at baseline (n = 161)*

Demographic measure	n (%)
Gender	
Women	130 (80.75)
Men	31 (19.25)
Age, mean ± SD, y	37.42 ± 10.53
BMI, mean ± SD, kg/m^2^	27.19 ± 7.10
Occupation	
Unemployed	82 (50.93)
Craft and related trades workers	4 (2.48)
Elementary occupations	2 (1.24)
Managers	6 (3.73)
Professional	16 (9.94)
Service and sales workers	6 (3.73)
Skilled agricultural, forestry and fishery workers	1 (0.62)
Technicians and associate professionals	5 (3.11)
Other occupations	26 (16.15)
Charlson Comorbidity Index	1.00 (1.00–2.00)

* BMI, body mass index.

**Table 2:** Tobacco, alcohol, and cannabis history of participants at baseline (n = 161)*

Demographic measure	n (%)
Tobacco status	
Never smoked	83 (51.55)
Former smoker	53 (32.92)
Current smoker	25 (15.53)
Lifetime tobacco consumption, median (IQR), pack‐years	8.00 (4.00–15.00)
Weekly alcohol consumption, median (IQR), units	0.00 (0.00–2.00)
Cannabis status	
Never used	61 (37.89)
Former user	20 (12.42)
Current user	80 (49.69)
Lifetime cannabis use, median (IQR), gram‐years	4.00 (1.00–15.00)

* IQR, interquartile range.

### CBMP details

The CBMP prescription details regarding dosage and administration can be found in Table 3. At baseline, 55.28% of patients (n = 89) were prescribed oil‐based CBMPs, 7.45% (n = 12) received dried flower, and 37.28% (n = 60) used both formulations. At 18 months, two patients (1.24%) were not receiving any CBMP prescription. Across all patients, the median dose of CBD was 25.00 (20.00–35.00) mg/24 hr, whereas the median dose of THC was 110.00 (10.00–135.00) mg/24 hr. The most frequently prescribed flos was EMT2 16%/<1% THC/CBD (Curaleaf International). EMC1 50/<4 mg/mL CBD/THC (Curaleaf International) and EMT 20 mg/mL THC (Curaleaf International) were the most frequently prescribed CBD‐ and THC‐dominant oils, respectively.

**Table 3:** Patient prescription information (n = 161)*

Prescription information	Baseline	1 month	3 months	6 months	12 months	18 months
Oils, n (%)	89 (55.28)	77 (47.83)	65 (40.37)	54 (33.54)	41 (25.47)	41 (25.47)
CBD, median (IQR), mg/24 h	20 (20.00–20.00)	20 (20.00–20.00)	20 (20.00–26.50)	20 (20.00–33.75)	20 (20.00–30.00)	20 (20.00–35.00)
THC, median (IQR), mg/24 h	1 (1.00–1.50)	5 (5.00–10.00)	8 (5.00–12.00)	10 (5.18–15.00)	10 (6.08–15.00)	11.6 (6.60–15.00)
Dried flower, n (%)	12 (7.45)	11 (6.83)	12 (7.45)	18 (1.18)	24 (14.91)	32 (19.88)
CBD, median (IQR), mg/24 h	1 (0.00–5.00)	5 (0.00–8.00)	10 (3.00–39.50)	9 (1.25–39.00)	18 (5.00–67.50)	15 (9.38–71.25)
THC, median (IQR), mg/24 h	20 (19.00–38.725)	100 (100.00–124.00)	102.50 (93.25–185.00)	141 (100.00–202.5)	200 (132.50–252.875)	198.75 (116.25–261.25)
Oils and dried flower, n (%)	60 (37.28)	73 (45.34)	84 (52.17)	85 (52.80)	92 (57.14)	86 (53.42)
CBD, median (IQR), mg/24 h	20 (17.9–21.00)	20 (20.00–25.00)	25 (20.00–50.00)	25 (20.00–50.00)	26.25 (15.00–65.00)	35 (20.00–70.00)
THC, median (IQR), mg/24 h	21 (20.00–22.00)	110 (100.00–113.00)	112 (105.54–200.00)	116.80 (107.50–212.50)	134 (110.00–215.00)	127.05 (109.63–222.50)
No prescription, n (%)	0 (0)	0 (0)	0 (0)	4 (2.48)	4 (2.48)	2 (1.24)

* CBD, cannabidiol; IQR, interquartile range; THC, (−)‐trans‐Δ^9^‐tetrahydrocannabinol.

### PROMs

Table 4 displays the changes between baseline and follow‐up scores for each of the PROMs as analyzed using a repeated‐measures ANOVA. The *t*‐tests with Bonferroni corrected *P* values for paired comparisons between baseline and the follow‐up intervals are shown in Supplementary Tables 1–17. There were improvements in pain across multiple modalities between baseline and all the subsequent time points as assessed by the SF‐MPQ‐2 and BPI (*P* < 0.010; Supplementary Tables 1–7). There was a decrease in the Pain‐VAS between baseline and 1, 6, 12, and 18 months (*P* < 0.050; Supplementary Table 8).

**Table 4:** One‐way repeated‐measures ANOVA statistical analysis of PROMs up until 18 months (n = 161)*

PROM	Baseline, mean ± SD	1 month, mean ± SD	3 months, mean ± SD	6 months, mean ± SD	12 months, mean ± SD	18 months, mean ± SD	F value	*P* value	η_p_^2^
SF‐MPQ‐2 affective descriptors subscale	5.02 ± 2.25	4.25 ± 2.45	4.24 ± 2.44	4.22 ± 2.52	4.27 ± 2.57	4.40 ± 2.44	9.326	<0.001	0.06
SF‐MPQ‐2 continuous pain subscale	6.03 ± 1.84	5.21 ± 2.11	5.18 ± 2.13	5.11 ± 2.05	5.30 ± 2.16	5.45 ± 2.10	15.81	<0.001	0.09
SF‐MPQ‐2 intermittent pain subscale	4.79 ± 2.16	4.17 ± 2.15	4.03 ± 2.22	4.05 ± 2.25	4.28 ± 2.37	4.33 ± 2.24	7.782	<0.001	0.05
SF‐MPQ‐2 neuropathic pain subscale	3.91 ± 2.3	3.37 ± 2.25	3.35 ± 2.22	3.42 ± 2.19	3.4 ± 2.36	3.46 ± 2.36	6.546	<0.001	0.04
SF‐MPQ‐2 total	4.94 ± 1.76	4.25 ± 1.95	4.2 ± 1.93	4.27 ± 2.05	4.31 ± 2.07	4.41 ± 1.95	13.38	<0.001	0.08
Pain‐VAS	6.90 ± 1.90	6.43 ± 1.93	6.56 ± 2.01	6.35 ± 2.08	6.33 ± 2.19	6.37 ± 2.01	4.589	<0.001	0.03
BPI interference score	6.96 ± 2.02	6.06 ± 2.06	6.02 ± 2.29	6.03 ± 2.27	6.17 ± 2.43	6.39 ± 2.19	14.51	<0.001	0.09
BPI severity score	5.87 ± 1.42	5.35 ± 1.51	5.34 ± 1.6	5.34 ± 1.65	5.33 ± 1.73	5.42 ± 1.62	9.708	<0.001	0.06
EQ‐5D‐5L mobility	3.08 ± 0.96	2.90 ± 0.97	2.84 ± 0.99	2.88 ± 1.03	2.84 ± 1.05	2.87 ± 1.05	5.072	<0.001	0.03
EQ‐5D‐5L self‐care	2.54 ± 1.01	2.35 ± 0.98	2.40 ± 1.05	2.38 ± 1.05	2.35 ± 1.01	2.40 ± 1.01	3.373	0.005	0.02
EQ‐5D‐5L usual activities	3.30 ± 0.99	2.89 ± 0.95	2.84 ± 1.02	2.90 ± 1.03	2.89 ± 1.07	2.96 ± 1.06	14.75	<0.001	0.08
EQ‐5D‐5L pain and discomfort	3.91 ± 0.79	3.36 ± 0.91	3.32 ± 0.91	3.34 ± 0.97	3.45 ± 0.96	3.50 ± 0.98	25.48	<0.001	0.14
EQ‐5D‐5L anxiety and depression	2.61 ± 1.23	2.31 ± 1.06	2.30 ± 1.06	2.26 ± 1.05	2.35 ± 1.11	2.39 ± 1.17	7.915	<0.001	0.05
EQ‐5D‐5L index value	0.25 ± 0.28	0.39 ± 0.27	0.40 ± 0.28	0.39 ± 0.30	0.37 ± 0.29	0.35 ± 0.30	25.05	<0.001	0.14
SQS	3.89 ± 2.33	5.28 ± 2.48	5.33 ± 4.26	5.30 ± 2.50	4.81 ± 2.59	4.46 ± 2.59	20.97	<0.001	0.12
GAD‐7	8.14 ± 6.29	6.33 ± 5.30	6.37 ± 5.37	6.30 ± 5.42	7.10 ± 6.05	7.22 ± 5.94	9.994	<0.001	0.06
PGIC	–	5.03 ± 1.45	5.18 ± 1.40	5.37 ± 1.40	5.35 ± 1.47	5.33 ± 1.43	5.726	<0.001	0.04

* The mean scores of the outcome measures with the SD of the mean are reported for each time point.

*** *P* < 0.001. ANOVA, analysis of variance; BPI, Brief Pain Inventory; EQ‐5D‐5L, five‐level EQ‐5D; GAD‐7, General Anxiety Disorder Seven‐Item Scale; Pain‐VAS, pain visual analog scale score; PGIC, Patient Global Impression of Change; PROM, patient‐reported outcome measure; SF‐MPQ‐2, Short‐Form McGill Pain Questionnaire 2; SQS, Single‐Item Sleep Quality Scale.

There was an increase between baseline and all the follow‐up intervals as evaluated by the EQ‐5D‐5L index value (*P* < 0.001; Supplementary Table 9). These improvements were indicated in the subscales of mobility at 12 and 18 months, usual activities at all time points, pain and discomfort at all time points, and anxiety and depression up until 18 months when compared to baseline (*P* < 0.001; Supplementary Tables 10–14). There were self‐reported improvements in the SQS from baseline to all time points and between 1, 3, and 6 months compared to 18 months (*P* < 0.001; Supplementary Table 15). There was a reduction in anxiety levels as reported by the GAD‐7 between baseline and all subsequent periods (*P* < 0.050; Supplementary Table 16). The PGIC analysis indicated individual improvements from 1 month to 6, 12, and 18 months (*P* < 0.010; Supplementary Table 17).

The MCID analysis of pain PROMs revealed clinically significant changes in the BPI severity score in a proportion of individuals at 1 month (n = 50; 31.06%), 3 months (*n* = 48; 29.81%), 6 months (*n* = 40; 24.84%), 12 months (*n* = 38; 23.60%), and 18 months (*n* = 29; 18.01%; Supplementary Table 18). Clinically significant changes were also reported in the BPI interference scale at 1 month (n = 68; 42.24%), 3 months (n = 62; 38.51%), 6 months (n = 58; 36.02%), 12 months (n = 50; 31.06%), and 18 months (n = 41; 25.47%). Finally, clinically significant changes were also reported by at least one‐quarter of individuals on the Pain‐VAS at 1 month (n = 64; 39.75%), 3 months (n = 54; 33.54%), 6 months (n = 60; 37.27%), 12 months (n = 41; 25.47%), and 18 months (n = 41; 25.47%). Additional proportions of patients who reported the MCID for other PROMs are detailed in Supplementary Table 18.

#### Logistic regression

Univariable logistic regression found that only one variable, CBD dosage >25.00 mg/day (OR 2.50, 95% CI 1.08–5.79, *P* = 0.032), was associated with an increase likelihood of experiencing a clinically significant improvement in pain severity on the BPI severity subscale (Supplementary Table 19). On multivariable logistic regression, being a former user of cannabis (OR 4.32, 95% CI 1.07–17.41, *P* = 0.040) was associated with an increased chance of reporting an MCID in pain severity in comparison to individuals who were cannabis naive (Supplementary Table 20). Being a current user (OR 2.96, 95% CI 0.89–9.83, *P* = 0.077) of cannabis at baseline was not associated with a change in the odds of reporting a clinically significant change in pain severity. CBD dosage >25.00 mg/day (OR 2.40, 95% CI 0.90–6.41, *P* = 0.081) was no longer associated with an MCID in pain severity on the BPI severity scale.

### AEs

The AEs reported by the patients are outlined in Table 5. A total of 50 patients (31.06%) reported one or more AEs with a total of 601 AEs (373.29%) documented. The most frequently occurring severity class was moderate (n = 258; 160.25%). Out of all the AEs, the most recorded were headache (n = 44; 27.33%), fatigue (n = 39; 24.22%), lethargy (n = 36, 22.36%), nausea (n = 33, 20.50%), and somnolence (n = 32; 19.88%). There were no incidents of life‐threatening /disabling AEs.

**Table 5:** Adverse events as reported by participants (n = 50)*

Adverse event	Mild	Moderate	Severe	Life‐threatening/disabling	Total, n (%)
Abdominal pain	7	10	1	0	18 (11.18)
Agitation	0	1	0	0	1 (0.62)
Amnesia	3	1	0	0	4 (2.48)
Anorexia	4	9	2	0	15 (9.32)
Arthralgia	0	1	0	0	1 (0.62)
Ataxia	6	8	1	0	15 (9.32)
Blurred vision	5	7	3	0	15 (9.32)
Cognitive disturbance	6	7	0	0	13 (8.07)
Concentration impairment	14	9	2	0	25 (15.53)
Confusion	7	1	2	0	10 (6.21)
Constipation	11	4	0	0	15 (9.32)
Cough	0	0	1	0	1 (0.62)
Delirium	3	2	0	0	5 (3.11)
Depression	1	0	0	0	1 (0.62)
Diarrhea	2	6	2	0	10 (6.21)
Dizziness	9	11	10	0	30 (18.63)
Dry eye	0	1	0	0	2 (1.24)
Dry mouth	26	3	0	0	29 (18.01)
Dysgeusia	4	1	0	0	5 (3.11)
Dyspnea	0	1	0	0	1 (0.62)
Fasciculation	1	0	0	0	1 (0.62)
Fall	1	4	0	0	5 (3.11)
Fatigue	7	17	15	0	39 (24.22)
Fever	8	1	0	0	9 (5.59)
Gastritis	0	1	0	0	1 (0.62)
Generalized muscle weakness	4	3	10	0	17 (10.56)
Headache	12	14	18	0	44 (27.33)
Hyperhidrosis	0	1	0	0	1 (0.62)
Insomnia	5	15	8	0	28 (17.39)
Irregular menstruation	1	0	0	0	1 (0.62)
Joint dislocation	0	0	1	0	1 (0.62)
Lethargy	14	22	0	0	36 (22.36)
Libido decreased	1	0	0	0	1 (0.62)
Lung infection	0	6	0	0	6 (3.73)
Mania	0	1	0	0	1 (0.62)
Nausea	19	12	2	0	33 (20.50)
Neuralgia	0	0	1	0	1 (0.62)
Pain	1	0	1	0	2 (1.24)
Palpitations	0	3	0	0	3 (1.86)
Pharyngitis	0	11	0	0	11 (6.83)
Rash NOS	3	1	0	0	4 (2.48)
Seizure	0	0	1	0	1 (0.62)
Sinus pain	1	0	0	0	1 (0.62)
Sinus tachycardia	0	1	0	0	1 (0.62)
Small intestine bacterial overgrowth	0	0	1	0	1 (0.62)
Somnolence	0	27	5	0	32 (19.88)
Spasticity	1	3	5	0	9 (5.59)
Tinnitus	0	1	0	0	1 (0.62)
Tremor	5	3	4	0	12 (7.45)
Upper respiratory infection	0	1	0	0	1 (0.62)
Urinary tract infection	0	3	1	0	4 (2.48)
Vasovagal reaction	0	0	1	0	1 (0.62)
Vertigo	11	6	5	0	22 (13.66)
Vomiting	8	2	1	0	11 (6.83)
Weight loss	10	4	0	0	14 (8.70)
Total, n (%)	238 (147.83)	258 (160.25)	99 (61.49)	0 (0)	601 (373.29)

* NOS, not otherwise specified.

## DISCUSSION

The present study evaluated the outcomes for individuals with HSD/hEDS prescribed CBMPs through the UKMCR. This case series found improvements in perceived pain severity and interference, general HRQoL, sleep quality, and anxiety in patients with HSD/hEDS after CBMP prescription. More than one‐quarter of individuals reported clinically significant changes in pain on the Pain‐VAS and BPI interference scale at 18 months. Meanwhile, 18.01% also reported a clinically significant change on the BPI severity score at 18 months. AEs were reported by 31.06% of the patient population, with a total incidence of 373.29%.

The observed improvements in pain are supported by previous literature on CBMPs for chronic pain management. Although there is limited high‐quality evidence directly supporting the present findings regarding pain relief for HSD/hEDS, the improvement in pain aligns with prior anecdotal and qualitative reports.^19^, ^20^ The study's results contribute to the existing body of literature investigating the use of CBMPs for chronic pain on a wider scale. The IASP reported a small beneficial effect of CBMPs on pain intensity in short‐ and long‐term investigations of unlicensed cannabis and nabiximols, respectively, but stated that the magnitude of the effect was small.^18^ Moreover, most evidence was produced by low‐quality studies, leading to a recommendation that there is insufficient evidence to conclude whether CBMPs are effective for pain.^18^ Another meta‐analysis by Wang et al^16^ focused on noninhaled CBMPs in individuals with follow‐up longer than four weeks. In this study, they modeled a 10% risk difference in reporting an MCID in pain intensity. Although there appears to be some concordance between these results, the challenge is comparing across the vast heterogeneity that exists in the CBMP literature. Randomized controlled trials in individuals treated for HSD/hEDS‐associated chronic pain, in particular, are limited.

In addition to improvements in pain‐specific metrics, patients with HSD/hEDS prescribed CBMPs reported improvements in EQ‐5D‐5L, SQS, and GAD‐7. This corresponds with improvement in HRQoL in patients with chronic pain reported previously through a cross‐sectional questionnaire study by Aviram et al.^19^ Assessment of EQ‐5D‐5L index value revealed an increase of 13% between one and six months in the patients with chronic pain.^19^ Moreover, a recent analysis of clinical outcomes across patients with a diverse spectrum of chronic pain conditions from the UKMCR also found an improvement in SQS, GAD‐7, and EQ‐5D‐5L indicators at 12 months.^22^ Benefits were observed across the measures after one month, with greater improvements following treatment with oils compared to flower.^22^ In the present study, changes were preserved up to 18 months of therapy. A meta‐analysis by Wang et al^16^ also reported a very small increase in the proportion of patients with improved physical functioning in patients prescribed noninhaled CBMPs. The review found no improvement in emotional functioning in patients taking CBMPs orally in comparison with placebo.^16^ However, they did find a small, yet statistically significant improvement in sleep quality.^16^ In the present study, improvements in both sleep quality and anxiety were reported. The difference between these findings may be attributable to inherent differences in study design between observational and randomized controlled trials. Moreover, they may be reflective of a more specific difference between individuals with HSD/hEDS compared to other chronic pain conditions or differences in the CBMPs prescribed. Finally, although anxiety and insomnia are common comorbidities in chronic pain, this cohort was being primarily treated for chronic pain secondary to HSD/hEDS.^1^, ^3^ Within this, more than one of seven participants reported a clinically significant improvement in symptoms of generalized anxiety and sleep quality at 18 months’ follow‐up.

This research represents one of the initial examinations into the AEs associated with the use of CBMPs in HSD/hEDS treatment up until 18 months. AEs were reported by 50 patients (31.06%). The total incidence of AEs reported by patients was 373.29%. AE incidence was relatively higher than previously reported in a UKMCR investigation into patients with chronic pain.^25^ Previous studies have suggested that women are associated with increased likelihood of AEs.^25^ This may also be secondary to the impact of central sensitization, a key mechanism in the development of chronic pain in HSD/hEDS.^51^ Central sensitization also underpins the development of fibromyalgia, which also had a similar prevalence of AEs in a prior UKMCR study.^52^ The most common AEs were headache (27.33%), fatigue (24.22%), lethargy (22.36%), nausea (20.50%), and somnolence (19.88%). There were no disabling or life‐threatening AEs reported. The AEs reported were not investigated further by clinicians, and so no causal relationship can be established with the CBMP treatment or other confounding factors.

The inherent limitation of observational studies is their susceptibility to confounding, which may misrepresent causal relationships between variables of interest. Therefore, causation cannot be inferred. Moreover, there is no comparator arm available. The placebo effects of medical cannabis could be enhanced secondary to psychoactive effects or anticipation of its efficacy, leading to an inflation in the reported effects.^53^, ^54^ Although PROMs are the current gold standard assessment for subjective effects of pain, future research should aim to include objective biomarkers to accompany results. For example, actigraphy or polysomnography would ideally be used to corroborate self‐reported sleep quality. This analysis was subject to attrition bias. To account for this and missing data, the BOCF method was used. This subsequently biases the results to the null. In the setting of an observational study embedded in a private health care clinic, there are additional factors that may influence the rate of attrition beyond survey nonresponse and lack of treatment efficacy, such as insufficient funds to continue treatment or movement between providers. The use of the BOCF method compounds over time with increasing attrition. It is important to complete randomized controlled trials with more complete follow‐up to examine whether the reduction in scores is secondary to the methods used to handle missing data or is secondary to factors such as pharmacological tolerance or lack of efficacy.

Furthermore, future analyses with larger sample sizes should aim to restrict heterogeneity by grouping patients by route of CBMP administration. With the implementation of observational techniques, the study cannot draw accurate conclusions on causation due to the lack of control over variations in prescription and THC/CBD concentration. The study is subject to selection bias because most patients analyzed were women.^55^ However, individuals with HSD/hEDS are more likely to be women, so this demographic split is representative of the wider patient population.^5^, ^6^ This may influence the reporting of AEs because women are more likely to report AEs, compared to men, across the biomedical literature.^56^ This study collected gender rather than sex, and this may contribute to sex‐specific differences in expression of cannabinoid receptors and response to phytocannabinoids.^55^ Most participants in this study were either past or current cannabis users, confounding the study further. Prior cannabis use may influence responses due to pharmacological tolerance to the effects of major cannabinoids or due to expectancy of the effects of cannabis on the body.^57^ A univariable and multivariable logistic regression was performed to understand the effects of confounding variables on the likelihood of reporting a clinically significant improvement in pain severity at 18 months. This found that being a previous user of cannabis was associated with an improved likelihood of reporting an improvement in pain severity. This may be due to selection bias, with previous users being more likely to know whether cannabis is effective for their symptoms compared to individuals who were cannabis naive. Current users of cannabis at baseline did not show the same effect. This in turn may be related to pharmacological tolerance due to repeated use. Although the use of multivariable logistic regression helps to control for confounding factors, it does not eliminate them, and randomized controlled trials in populations who were cannabis naive are necessary to determine the true effects of CBMPs.

This study reports an association between CBMP treatment and reported improvements in pain and HRQoL among patients with HSD/hEDS. At 18 months, between 18.01% and 25.47% of individuals reported a clinically significant improvement in their pain depending on the assessment measure used. Because CBMPs may only be prescribed for UK patients when they have failed licensed treatments, this may represent an acceptable chance of achieving improvement in chronic pain for these patients. CBMPs were well tolerated by many patients, with most reporting no adverse effects (68.94%). However, due to the limitations of study design, it is not possible to prove a causative association between CBMPs and the outcomes. Ultimately, these findings may help guide current clinical practice and shared decision‐making between patients and physicians; high‐quality randomized controlled trials will be necessary to understand the true efficacy and safety of CBMPs for people with chronic pain secondary to HSD/hEDS.

## AUTHOR CONTRIBUTIONS

All authors contributed to at least one of the following manuscript preparation roles: conceptualization AND/OR methodology, software, investigation, formal analysis, data curation, visualization, and validation AND drafting or reviewing/editing the final draft. As corresponding author, Dr Sodergren confirms that all authors have provided the final approval of the version to be published, and takes responsibility for the affirmations regarding article submission (eg, not under consideration by another journal), the integrity of the data presented, and the statements regarding compliance with institutional review board/Declaration of Helsinki requirements.

## Supporting information


**Disclosure form**.

**Appendix S1:** Supplementary Material 1.

**Appendix S2:** Supplementary Material 2.
