# Supplementary material for: UK Medical Cannabis Registry: An Analysis of Outcomes of Medical Cannabis Therapy for Hypermobility‐Associated Chronic Pain
Source: ACR Open Rheumatol. 2025 Mar 13;7(3):e70024. doi: 10.1002/acr2.70024 (PMC11905011; doi:10.1002/acr2.70024)
Supplement: Supplementary file 3 — Appendix S2: Supplementary Material 2. [file ACR2-7-e70024-s002.docx]

**Supplementary Material**

**Supplementary Table 1**

| **Follow-up Interval** | **Mean ± SD, *p-*value** | | | | | | | | | |
| --- | --- | --- | --- | --- | --- | --- | --- | --- | --- | --- |
|  | **Baseline** | **1** | **3** | | **6** | | **12** | | **18** | |
| Baseline |  |  |  |  | |  | |  | |  |
| 1 | -.76 ± 1.69, *p*<.001 |  |  |  | |  | |  | |  |
| 3 | -.78 ± 1.86, *p*<.001 | -.02 ± 1.83, *p* = 1 |  |  | |  | |  | |  |
| 6 | -.8 ± 1.94, *p<*.001 | -.03 ± 1.96, *p* = 1 | -.02 ± 1.66, *p* = 1 |  | |  | |  | |  |
| 12 | -.74 ± 1.56, *p*<.001 | -.02 ± 1.88, *p* = 1 | .04 ± 1.71, *p* = 1 | .06 ± 1.73, *p* = 1 | |  | |  | |  |
| 18 | -.61 ± 1.42, *p*<.001 | -.15 ± 2.03, *p* = 1 | .16 ± 1.89, *p* = 1 | .19 ± 1.99, *p* = 1 | | .13 ± 1.47, *p* = 1 | |  | |  |

*Pairwise comparison statistical analysis of SF-MPQ-2 Affective Descriptors Subscale up until 18 months (n = 156)*

**Supplementary Table 2**

*Pairwise comparison statistical analysis of SF-MPQ-2 Continuous Pain Subscale up until 18 months (n = 156)*

| **Follow-up Interval** | **Mean ± SD, *p-*value** | | | | | | | | | |
| --- | --- | --- | --- | --- | --- | --- | --- | --- | --- | --- |
|  | **Baseline** | **1** | **3** | | **6** | | **12** | | **18** | |
| Baseline |  |  |  |  | |  | |  | |  |
| 1 | -.82 ± 1.37, *p*<.001 |  |  |  | |  | |  | |  |
| 3 | -.86 ± 1.44, *p*<.001 | -.03 ± 1.49, *p* = 1 |  |  | |  | |  | |  |
| 6 | -.92 ± 1.56, *p*<.001 | .09 ± 1.62, *p* = 1 | -.06 ± 1.48, *p* = 1 |  | |  | |  | |  |
| 12 | -.73 ± 1.45, *p*<.001 | .1 ± 1.51, *p* = 1 | .13 ± 1.43, *p* = 1 | .19 ± 1.35, *p* = .499 | |  | |  | |  |
| 18 | -.58 ± 1.42, *p*<.001 | .25 ± 1.72, *p* = .458 | .28 ± 1.68, *p* = .245 | .34 ± 1.61, *p* = .059 | | .15 ± 1.47, *p* = 1 | |  | |  |

**Supplementary Table 3**

*Pairwise comparison statistical analysis of SF-MPQ-2 Intermittent Pain Subscale up until 18 months (n = 156)*

| **Follow-up Interval** | **Mean ± SD, *p-*value** | | | | | | | | | |
| --- | --- | --- | --- | --- | --- | --- | --- | --- | --- | --- |
|  | **Baseline** | **1** | **3** | | **6** | | **12** | | **18** | |
| Baseline |  |  |  |  | |  | |  | |  |
| 1 | -.62 ± 1.85, *p*<.001 |  |  |  | |  | |  | |  |
| 3 | -.76 ± 1.68, *p*<.001 | -.14 ± 1.62, *p* = 1 |  |  | |  | |  | |  |
| 6 | -.74 ± 1.96, *p*<.001 | -.12 ± 1.81, *p* = 1 | .02 ± 1.6, *p* = 1 |  | |  | |  | |  |
| 12 | -.51 ± 1.65, *p* = .001 | .11 ± 1.85, *p* = 1 | .25 ± 1.7, *p* = .406 | .23 ± 1.75, *p* = .624 | |  | |  | |  |
| 18 | -.46 ± 1.68, *p* = .005 | .16 ± 2.03, *p* = 1 | .3 ± 1.83, *p* = .259 | .28 ± 1.87, *p* = .396 | | .04 ± 1.5, *p* = 1 | |  | |  |

**Supplementary Table 4**

*Pairwise comparison statistical analysis of SF-MPQ-2 Neuropathic Pain Subscale up until 18 months (n = 156)*

| **Follow-up Interval** | **Mean ± SD, *p-*value** | | | | | | | | | |
| --- | --- | --- | --- | --- | --- | --- | --- | --- | --- | --- |
|  | **Baseline** | **1** | **3** | | **6** | | **12** | | **18** | |
| Baseline |  |  |  |  | |  | |  | |  |
| 1 | -.54 ± 1.51, *p*<.001 |  |  |  | |  | |  | |  |
| 3 | -.56 ± 1.48, *p*<.001 | -.02 ± 1.31, *p* = 1 |  |  | |  | |  | |  |
| 6 | -.49 ± 1.64, *p* = .002 | .05 ± 1.36, *p* = 1 | .07 ± 1.25, *p* = 1 |  | |  | |  | |  |
| 12 | -.51 ± 1.34, *p*<.001 | .03 ± 1.44, *p* = 1 | .05 ± 1.34, *p* = 1 | -.02 ± 1.44, *p* = 1 | |  | |  | |  |
| 18 | -.45 ± 1.44, *p*<.001 | .09 ± 1.79, *p* = 1 | .11 ± 1.58, *p* = 1 | .04 ± 1.54, *p* = 1 | | .06 ± 1.3, *p* = 1 | |  | |  |

**Supplementary Table 5**

*Pairwise comparison statistical analysis of SF-MPQ-2 Total up until 18 months (n = 156)*

| **Follow-up Interval** | **Mean ± SD, *p-*value** | | | | | | | | | |
| --- | --- | --- | --- | --- | --- | --- | --- | --- | --- | --- |
|  | **Baseline** | **1** | **3** | | **6** | | **12** | | **18** | |
| Baseline |  |  |  |  | |  | |  | |  |
| 1 | -.69 ± 1.22, *p*<.001 |  |  |  | |  | |  | |  |
| 3 | -.74 ± 1.26, *p*<.001 | -.05 ± 1.24, *p* = 1 |  |  | |  | |  | |  |
| 6 | -.66 ± 1.45, *p*<.001 | .02 ± 1.46, *p* = 1 | .07 ± 1.29, *p* = 1 |  | |  | |  | |  |
| 12 | -.62 ± 1.2, *p*<.001 | .07 ± 1.34, *p* = 1 | .12 ± 1.24, *p* = 1 | .04 ± 1.2, *p* = 1 | |  | |  | |  |
| 18 | -.53 ± 1.27, *p*<.001 | .16 ± 1.59, *p* = 1 | .21 ± 1.4, *p* = .356 | .14 ± 1.4, *p* = 1 | | .1 ± 1.2, *p* = 1 | |  | |  |

**Supplementary Table 6**

*Pairwise comparison statistical analysis of PAIN-VAS up until 18 months (n = 156)*

| **Follow-up Interval** | **Mean ± SD, *p-*value** | | | | | | | | | |
| --- | --- | --- | --- | --- | --- | --- | --- | --- | --- | --- |
|  | **Baseline** | **1** | **3** | | **6** | | **12** | | **18** | |
| Baseline |  |  |  |  | |  | |  | |  |
| 1 | -.47 ± 1.86, *p* = .012 |  |  |  | |  | |  | |  |
| 3 | -.33 ± 1.78, *p* = .123 | .13 ± 1.8, *p* = 1 |  |  | |  | |  | |  |
| 6 | -.54 ± 1.54, *p*<.001 | -.08 ± 1.93, *p* = 1 | -.21 ± 1.77, *p* = .831 |  | |  | |  | |  |
| 12 | -.57 ± 1.76, *p*<.001 | -.1 ± 2.06, *p* = 1 | -.24 ± 1.74, *p* = .542 | -.03 ± 1.67, *p* = 1 | |  | |  | |  |
| 18 | -.53 ± 1.47, *p*<.001 | -.06 ± 2.07, *p* = 1 | -.19 ± 1.95, *p* = 1 | .2 ± 1.5, *p* = 1 | | .04 ± 1.75, *p* = 1 | |  | |  |

**Supplementary Table 7**

*Pairwise comparison statistical analysis of BPI Interference Score up until 18 months (n = 157)*

| **Follow-up Interval** | **Mean ± SD, *p-*value** | | | | | | | | | |
| --- | --- | --- | --- | --- | --- | --- | --- | --- | --- | --- |
|  | **Baseline** | **1** | **3** | | **6** | | **12** | | **18** | |
| Baseline |  |  |  |  | |  | |  | |  |
| 1 | -.9 ± 1.49, *p* <.001 |  |  |  | |  | |  | |  |
| 3 | -.94 ± 1.79, *p* <.001 | -.04 ± 1.75, *p* = 1 |  |  | |  | |  | |  |
| 6 | -.93 ± 1.77, *p*<.001 | -.03 ± 1.75, *p* = 1 | .01 ± 1.63, *p* = 1 |  | |  | |  | |  |
| 12 | -.8 ± 1.72, *p*<.001 | .11 ± 1.88, *p* = 1 | .14 ± 1.94, *p* = 1 | .14 ± 1.52, *p* = 1 | |  | |  | |  |
| 18 | -.57 ± 1.3, *p*<.001 | .33 ± 1.8, *p* = .129 | .37 ± 1.84, *p* = .076 | .36 ± 1.7, *p* = .051 | | .23 ± 1.46, *p* = .322 | |  | |  |

**Supplementary Table 8**

*Pairwise comparison statistical analysis of BPI Severity Score up until 18 months (n = 157)*

| **Follow-up Interval** | **Mean ± SD, *p-*value** | | | | | | | | | |
| --- | --- | --- | --- | --- | --- | --- | --- | --- | --- | --- |
|  | **Baseline** | **1** | **3** | | **6** | | **12** | | **18** | |
| Baseline |  |  |  |  | |  | |  | |  |
| 1 | -.52 ± 1.19, *p* <.001 |  |  |  | |  | |  | |  |
| 3 | -.53 ± 1.13, *p* <.001 | -.01 ± 1.24, *p* = 1 |  |  | |  | |  | |  |
| 6 | -.53 ± 1.16, *p*<.001 | -.01 ± 1.33, *p* = 1 | 0 ± 1, *p* = 1 |  | |  | |  | |  |
| 12 | -.54 ± 1.19, *p*<.001 | -.02 ± 1.44, *p* = 1 | -.01 ± 1.11, *p* = 1 | -.01 ± 1.16, *p* = 1 | |  | |  | |  |
| 18 | -.45 ± 1.08, *p*<.001 | .07 ± 1.42, *p* = 1 | .08 ± 1.26, *p* = 1 | .08 ± 1.17, *p* = 1 | | .09 ± 1.04, *p* = 1 | |  | |  |

**Supplementary Table 9**

*Pairwise comparison statistical analysis of EQ-5D-5L Mobility up until 18 months (n = 161)*

| **Follow-up Interval** | **Mean ± SD, *p-*value** | | | | | | | | | |
| --- | --- | --- | --- | --- | --- | --- | --- | --- | --- | --- |
|  | **Baseline** | **1** | **3** | | **6** | | **12** | | **18** | |
| Baseline |  |  |  |  | |  | |  | |  |
| 1 | -.18 ± .71, *p* = .009 |  |  |  | |  | |  | |  |
| 3 | -.24 ± .8, *p* = .002 | -.06 ± .74, *p* = 1 |  |  | |  | |  | |  |
| 6 | -.2 ± .71, *p* = .003 | -.02 ± .76, *p* = 1 | .04 ± .71, *p* = 1 |  | |  | |  | |  |
| 12 | -.24 ± .7, *p*<.001 | -.06 ± .74, *p* = 1 | -.01 ± .69, *p* = 1 | -.04 ± .61, *p* = 1 | |  | |  | |  |
| 18 | -.21 ± .59, *p*<.001 | -.03 ± .77, *p* = 1 | .02 ± .84, *p* = 1 | .02 ± .84, *p* = 1 | | .03 ± .67, *p* = 1 | |  | |  |

**Supplementary Table 10**

*Pairwise comparison statistical analysis of EQ-5D-5L Selfcare up until 18 months (n = 161)*

| **Follow-up Interval** | **Mean ± SD, *p-*value** | | | | | | | | | |
| --- | --- | --- | --- | --- | --- | --- | --- | --- | --- | --- |
|  | **Baseline** | **1** | **3** | | **6** | | **12** | | **18** | |
| Baseline |  |  |  |  | |  | |  | |  |
| 1 | -.19 ± .7, *p* = .006 |  |  |  | |  | |  | |  |
| 3 | -.14 ± .75, *p* = .128 | .05 ± .68, *p* = 1 |  |  | |  | |  | |  |
| 6 | -.16 ± .69, *p* = .02 | .02 ± .77, *p* = 1 | -.02 ± .68, *p* = 1 |  | |  | |  | |  |
| 12 | -.19 ± .64, *p* = .002 | 0 ± .72, *p* = 1 | .02 ± .67, *p* = 1 | -.02 ± .68, *p* = 1 | |  | |  | |  |
| 18 | -.14 ± .56, *p* = .008 | .04 ± .74, *p* = 1 | -.01 ± .69, *p* = 1 | .02 ± .84, *p* = 1 | | .04 ± .53, *p* = 1 | |  | |  |

**Supplementary Table 11**

*Pairwise comparison statistical analysis of EQ-5D-5L Usual Activities up until 18 months (n = 161)*

| **Follow-up Interval** | **Mean ± SD, *p-*value** | | | | | | | | | |
| --- | --- | --- | --- | --- | --- | --- | --- | --- | --- | --- |
|  | **Baseline** | **1** | **3** | | **6** | | **12** | | **18** | |
| Baseline |  |  |  |  | |  | |  | |  |
| 1 | -.42 ± .79, *p*<.001 |  |  |  | |  | |  | |  |
| 3 | -.47 ± .84, *p*<.001 | -.05 ± .84, *p* = 1 |  |  | |  | |  | |  |
| 6 | -.4 ± .75, *p*<.001 | .01 ± .81, *p* = 1 | .06 ± .68, *p* = 1 |  | |  | |  | |  |
| 12 | -.42 ± .8, *p*<.001 | 0 ± .87, *p* = 1 | .05 ± .76, *p* = 1 | -.01 ± .71, *p* = 1 | |  | |  | |  |
| 18 | -.34 ± .79, *p*<.001 | .07 ± .89, *p* = 1 | .12 ± .9, *p* = .49 | .06 ± .82, *p* = 1 | | .07 ± .73, *p* = 1 | |  | |  |

**Supplementary Table 12**

*Pairwise comparison statistical analysis of EQ-5D-5L Pain and Discomfort up until 18 months (n = 161)*

| **Follow-up Interval** | **Mean ± SD, *p-*value** | | | | | | | | | |
| --- | --- | --- | --- | --- | --- | --- | --- | --- | --- | --- |
|  | **Baseline** | **1** | **3** | | **6** | | **12** | | **18** | |
| Baseline |  |  |  |  | |  | |  | |  |
| 1 | -.55 ± .85, *p*<.001 |  |  |  | |  | |  | |  |
| 3 | -.59 ± .8, *p*<.001 | -.04 ± .75, *p* = 1 |  |  | |  | |  | |  |
| 6 | -.57 ± .81, *p*<.001 | -.02 ± .82, *p* = 1 | .02 ± .66, *p* = 1 |  | |  | |  | |  |
| 12 | -.46 ± .73, *p*<.001 | .09 ± .88, *p* = 1 | .13 ± .75, *p* = .174 | .11 ± .64, *p* = .172 | |  | |  | |  |
| 18 | -.41 ± .77, *p*<.001 | .14 ± .97, *p* = .387 | .18 ± .9, *p* = .073 | .16 ± .8, *p* = .067 | | .05 ± .63, *p* = 1 | |  | |  |

**Supplementary Table 13**

*Pairwise comparison statistical analysis of EQ-5D-5L Anxiety and Depression up until 18 months (n = 161)*

| **Follow-up Interval** | **Mean ± SD, *p-*value** | | | | | | | | | |
| --- | --- | --- | --- | --- | --- | --- | --- | --- | --- | --- |
|  | **Baseline** | **1** | **3** | | **6** | | **12** | | **18** | |
| Baseline |  |  |  |  | |  | |  | |  |
| 1 | -.3 ± .83, *p*<.001 |  |  |  | |  | |  | |  |
| 3 | -.31 ± 1, *p*<.001 | -.01 ± .79, *p* = 1 |  |  | |  | |  | |  |
| 6 | -.35 ± .85, *p*<.001 | -.05 ± .69, *p* = 1 | -.04 ± .75, *p* = 1 |  | |  | |  | |  |
| 12 | -.27 ± .83, *p*<.001 | .04 ± .77, *p* = 1 | .04 ± .82, *p* = .174 | .09 ± .64, *p* = .509 | |  | |  | |  |
| 18 | -.22 ± .87, *p* = .008 | .08 ± .86, *p* = 1 | .09 ± .94, *p* = 1 | .13 ± .76, *p* = .185 | | .04 ± .67, *p* = 1 | |  | |  |

**Supplementary Table 13**

*Pairwise comparison statistical analysis of EQ-5D-5L Index Values up until 18 months (n = 161)*

| **Follow-up Interval** | **Mean ± SD, *p-*value** | | | | | | | | | |
| --- | --- | --- | --- | --- | --- | --- | --- | --- | --- | --- |
|  | **Baseline** | **1** | **3** | | **6** | | **12** | | **18** | |
| Baseline |  |  |  |  | |  | |  | |  |
| 1 | .15 ± .23, *p*<.001 |  |  |  | |  | |  | |  |
| 3 | .15 ± .24, *p*<.001 | 0 ± .2, *p* = 1 |  |  | |  | |  | |  |
| 6 | .14 ± .22, *p*<.001 | -.01 ± .2, *p* = 1 | -.01 ± .16, *p* = 1 |  | |  | |  | |  |
| 12 | .13 ± .21, *p*<.001 | -.02 ± .23, *p* = 1 | -.02 ± .2, *p* = .653 | -.02 ± .16, *p* = 1 | |  | |  | |  |
| 18 | .1 ± .21, *p*<.001 | -.04 ± .24, *p* = .109 | -.05 ± .22, *p* = .043 | -.04 ± .2, *p* = .075 | | -.02 ± .15, *p* = .328 | |  | |  |

**Supplementary Table 14**

*Pairwise comparison statistical analysis of SQS up until 18 months (n = 160)*

| **Follow-up Interval** | **Mean ± SD, *p-*value** | | | | | | | | | |
| --- | --- | --- | --- | --- | --- | --- | --- | --- | --- | --- |
|  | **Baseline** | **1** | **3** | | **6** | | **12** | | **18** | |
| Baseline |  |  |  |  | |  | |  | |  |
| 1 | 1.39 ± 2.42, *p*<.001 |  |  |  | |  | |  | |  |
| 3 | 1.44 ± 2.46, *p*<.001 | .04 ± 2.09, *p* = 1 |  |  | |  | |  | |  |
| 6 | 1.41 ± 2.38, *p*<.001 | .02 ± 2.25, *p* = 1 | -.03 ± 2.11, *p* = 1 |  | |  | |  | |  |
| 12 | .93 ± 2.08, *p*<.001 | -.47 ± 2.45, *p* = .101 | -.51 ± 2.33, *p* = .036 | -.49 ± 2.09, *p* = .022 | |  | |  | |  |
| 18 | .57 ± 1.82, *p*<.001 | -.83 ± 2.63, *p*<.001 | -.87 ± 2.58, *p*<.001 | -.84 ± 2.31, *p*<.001 | | -.36 ± 1.96, *p* = .135 | |  | |  |

**Supplementary Table 15**

*Pairwise comparison statistical analysis of GAD-7 up until 18 months (n = 161)*

| **Follow-up Interval** | **Mean ± SD, *p-*value** | | | | | | | | | |
| --- | --- | --- | --- | --- | --- | --- | --- | --- | --- | --- |
|  | **Baseline** | **1** | **3** | | **6** | | **12** | | **18** | |
| Baseline |  |  |  |  | |  | |  | |  |
| 1 | -1.81 ± 4.48, *p*<.001 |  |  |  | |  | |  | |  |
| 3 | -1.77 ± 5, *p*<.001 | .04 ± 3.36, *p* = 1 |  |  | |  | |  | |  |
| 6 | -1.84 ± 4.49, *p*<.001 | -.02 ± 3.53, *p* = 1 | -.07 ± 3.3, *p* = 1 |  | |  | |  | |  |
| 12 | -1.04 ± 4.39, *p* = .018 | .77 ± 4.34, *p* = .154 | .73 ± 4.45, *p* = .239 | .8 ± 3.48, *p* = .026 | |  | |  | |  |
| 18 | -.93 ± 3.77, *p*= .013 | .89 ± 4.45, *p* = .073 | -.84 ± 4.54, *p* = .117 | .91 ± 4.04, *p* = .028 | | -.12 ± 3.82, *p* = 1 | |  | |  |

**Supplementary Table 16**

*Pairwise comparison statistical analysis of PGIC up until 18 months (n = 137)*

| **Follow-up Interval** | **Mean ± SD, *p-*value** | | | | | | | | | |
| --- | --- | --- | --- | --- | --- | --- | --- | --- | --- | --- |
|  | **Baseline** | **1** | **3** | | **6** | | **12** | | **18** | |
| Baseline |  |  |  |  | |  | |  | |  |
| 1 |  |  |  |  | |  | |  | |  |
| 3 |  | .15 ± 1, *p* = .532 |  |  | |  | |  | |  |
| 6 |  | .34 ± 1.03, *p*<.001 | .2 ± .95, *p* = .096 |  | |  | |  | |  |
| 12 |  | .32 ± 1.13, *p* = .007 | .18 ± 1.13, *p* = .431 | -.02 ± .93, *p* = 1 | |  | |  | |  |
| 18 |  | .3 ± .88, *p*<.001 | .15 ± 1.11, *p* = .651 | -.04 ± .98, *p* = 1 | | -.02 ± .95, *p* = 1 | |  | |  |

**Supplementary Table 17**

*Minimal clinically important difference analysis against baseline of Pain-VAS and BPI (n = 161)*

| **PROM** | **% of MCID** | | | | | | | | | |
| --- | --- | --- | --- | --- | --- | --- | --- | --- | --- | --- |
|  | **1** | | **3** | | **6** | | **12** | | **18** | |
| Pain-VAS | 20.5% | 21.74% | | 13.66% | | 12.42% | | 8.07% | |  |
| BPI Interference Score | 9.32% | 11.18% | | 8.7% | | 5.59% | | 4.35% | |  |
| BPI Severity Score | 10.56% | 9.94% | | 8.07% | | 6.21% | | 4.97% | |  |
